# Barth syndrome: an **X**-linked cause of fetal cardiomyopathy and stillbirth

**DOI:** 10.1002/pd.2599

**Published:** 2010-10

**Authors:** C G Steward, R A Newbury-Ecob, R Hastings, S F Smithson, B Tsai-Goodman, O W Quarrell, W Kulik, R Wanders, M Pennock, M Williams, J L Cresswell, I L Gonzalez, P Brennan

**Affiliations:** 1 Department of Paediatric Haematology, Oncology & BMT, Royal Hospital for Children, Upper Maudlin StBristol, BS2 8BJ & Department of Cellular & Molecular Medicine, School of Medical Sciences, University WalkBristol, BS8 1TD, UK; 2 Department of Clinical Genetics, St Michael's HospitalSouthwell Street, Bristol, BS2 8EG, UK; 3 Department of Paediatric Cardiology, Royal Hospital for ChildrenUpper Maudlin St, Bristol, BS2 8BJ, UK; 4 Sheffield Clinical Genetics Service, Sheffield Children's HospitalWestern Bank, Sheffield, S10 2TH, UK; 5 Department of Clinical Chemistry, Laboratory Genetic Metabolic Diseases, University of AmsterdamAmsterdam, The Netherlands; 6 Bristol Genetics Laboratory, Southmead HospitalBristol, BS10 5NB, UK; 7 Department of Obstetrics & Gynaecology, Chesterfield Royal HospitalCalow, Chesterfield, S44 5BL, UK; 8 Molecular Diagnostics Laboratory, Nemours Biomedical Research, Alfred I. duPont Hospital for ChildrenWilmington, Delaware 19899, USA; 9 Teesside Genetics Unit, Northern Genetics Service, The James Cook University HospitalMarton Road, Middlesbrough, TS4 3BW, UK

**Keywords:** Barth syndrome, fetal, hydrops, neonatal, perinatal

## Abstract

**Objective:**

Barth Syndrome (BTHS) is an X-linked multisystem disorder (OMIM 302060) usually diagnosed in infancy and characterized by cardiac problems [dilated cardiomyopathy (DCM) ± endocardial fibroelastosis (EFE) ± left ventricular non-compaction (LVNC)], proximal myopathy, feeding problems, growth retardation, neutropenia, organic aciduria and variable respiratory chain abnormalities. We wished to determine whether BTHS had a significant impact on fetal and perinatal health in a large cohort of family groups originating from a defined region.

**Method:**

Case note review on 19 families originating from the UK and known to the Barth Syndrome Service of the Bristol Royal Hospital for Children.

**Results:**

Details are presented on six kindreds (32%) with genetically and biochemically proven BTHS that demonstrate a wider phenotype including male fetal loss, stillbirth and severe neonatal illness or death. In these families, 9 males were stillborn and 14 died as neonates or infants but there were no losses of females. BTHS was definitively proven in five males with fetal onset of DCM ± hydrops/EFE/LVNC.

**Conclusion:**

These findings stress the importance of considering BTHS in the differential diagnosis of unexplained male hydrops, DCM, EFE, LVNC or pregnancy loss, as well as in neonates with hypoglycemia, lactic acidosis and idiopathic mitochondrial disease. Copyright © 2010 John Wiley & Sons, Ltd.

## INTRODUCTION

Barth Syndrome (BTHS) is an X-linked disease conventionally characterized by dilated cardiomyopathy (DCM) with endocardial fibroelastosis (EFE), skeletal (predominantly proximal) myopathy, growth retardation, neutropenia and organic aciduria [especially excess of 3-methylglutaconic (3-MGC) acid] (Kelley *et al.*, 1991; Barth *et al.*, 1999; Barth, 2005). It results from mutations of the gene *TAZ* (previously termed tafazzin), located at Xq28, which encodes a highly conserved acyltransferase (Bione *et al.*, 1996).

Understanding the pathogenesis of BTHS has so far remained elusive but one major consequence of deficiency of this enzyme is defective remodelling of phospholipid side chains (Vreken *et al.*, 2000). This results in deficiency of cardiolipin (CL) with four linoleic acid side chains and relative excess of monolysocardiolipin (MLCL, with just three side chains), and hence to a highly abnormal MLCL/CL ratio (Valianpour *et al.*, 2005; Schlame, 2007). This feature has recently allowed the development of a highly sensitive and specific assay applicable to lymphocytes, platelets, muscle biopsies, fibroblasts or even single stored neonatal bloodspots (Kulik *et al.*, 2008).

Although first reported in 1983 (Barth *et al.*, 1983), relatively few children have been diagnosed and still only 160 unrelated cases are known to the Barth Syndrome Foundation genetic database for the disorder (http://www.barthsyndrome.org/). Barriers to case ascertainment have been that (1) the relatively small increase in organic acid excretion is easily missed or may even be absent (Schmidt *et al.*, 2004), (2) neutropenia may be intermittent (Barth *et al.*, 1999) or non-existent (manuscript in preparation), and (3) a viral etiology for acute DCM is often assumed when this is seen in combination with neutropenia. The latter misdiagnosis is compounded by often remarkable improvements in the cardiomyopathy with age, confirming a suspicion that the patient has recovered from an acute viral insult.

However, recent years have seen rapid increases in the rate of case identification, driven by a combination of the realization of the wide and variable phenotype, and the introduction of genetic testing and CL assays (Cantlay *et al.*, 1999; Gonzalez, 2005; Houtkooper *et al.*, 2009). Features of the disease which are less well known include: hypertrophic cardiomyopathy, isolated left ventricular non-compaction (LVNC), ventricular arrhythmia (Bleyl *et al.*, 1997; Ichida *et al.*, 2001; Chen *et al.*, 2002; Brady *et al.*, 2006; Spencer *et al.*, 2006), motor delay, poor appetite, fatigue and exercise intolerance, hypoglycemia, lactic acidosis, hyperammonemia and dramatic late catch-up growth after growth delay throughout childhood (Spencer *et al.*, 2005; Spencer *et al.*, 2006; Spencer *et al.*, 2007; Yen *et al.*, 2008).

CL comprises approximately one quarter of all mitochondrial phospholipid and this probably explains the observation of abnormal mitochondrial structure and minor abnormalities of respiratory chain function assays in some patients (Neustein *et al.*, 1979; Barth *et al.*, 1996; Xu *et al.*, 2005; Acehan *et al.*, 2007). BTHS is, therefore, a unique form of mitochondrial disease where the membrane structural perturbation due to abnormal phospholipid composition interferes with the mitochondrial function. Patients frequently have profound proximal myopathy and exercise intolerance (Spencer *et al.*, 2007). The cardiomyopathy is usually dilated at presentation but can swing between dilated and hypertrophic. Ventricular arrhythmia may occur, especially during adolescence, and may result in sudden death (Spencer *et al.*, 2005).

Several previous reports have shown that BTHS can also cause fetal cardiomyopathy. Two papers (Cardonick *et al.*, 1997; Brady *et al.*, 2006) have reported families with strong histories of male DCM or male infant/toddler death, which was finally ascribed to BTHS. Fetuses from each report showed ventricular dysfunction, cardiomegaly or ascites on ultrasound at 32 to 33 weeks' gestation. A subsequent pregnancy in one of these families was terminated electively at 18 weeks' gestation after chorionic villus biopsy and amniocentesis confirmed an affected male (Brady *et al.*, 2006). Autopsy even at this early stage of fetal life demonstrated cardiomegaly, EFE and subendocardial vacuolization of the myocytes.

These findings, accompanied by an excess of late spontaneous abortions and stillbirths of male fetuses in families attending the BTHS clinics at the Bristol Royal Hospital for Children, led us to undertake a detailed analysis of fetal deaths in affected UK families. These confirm that BTHS is a definite cause of fetal loss in some families and raise suspicions that it may cause miscarriage at all phases of pregnancy.

## METHODS

Information on fetal and childhood deaths or cardiac problems was collated from case note review, clinic interviews and further information on detailed family history submitted by parents to the UK Barth Syndrome Service at the Bristol Royal Hospital for Children. All families were Caucasian and of UK ancestry. BTHS was proven biochemically (by assay of MLCL/CL ratio) and genetically (by *TAZ* gene sequencing) in at least one member of each kindred.

## RESULTS

**Figure 1:**
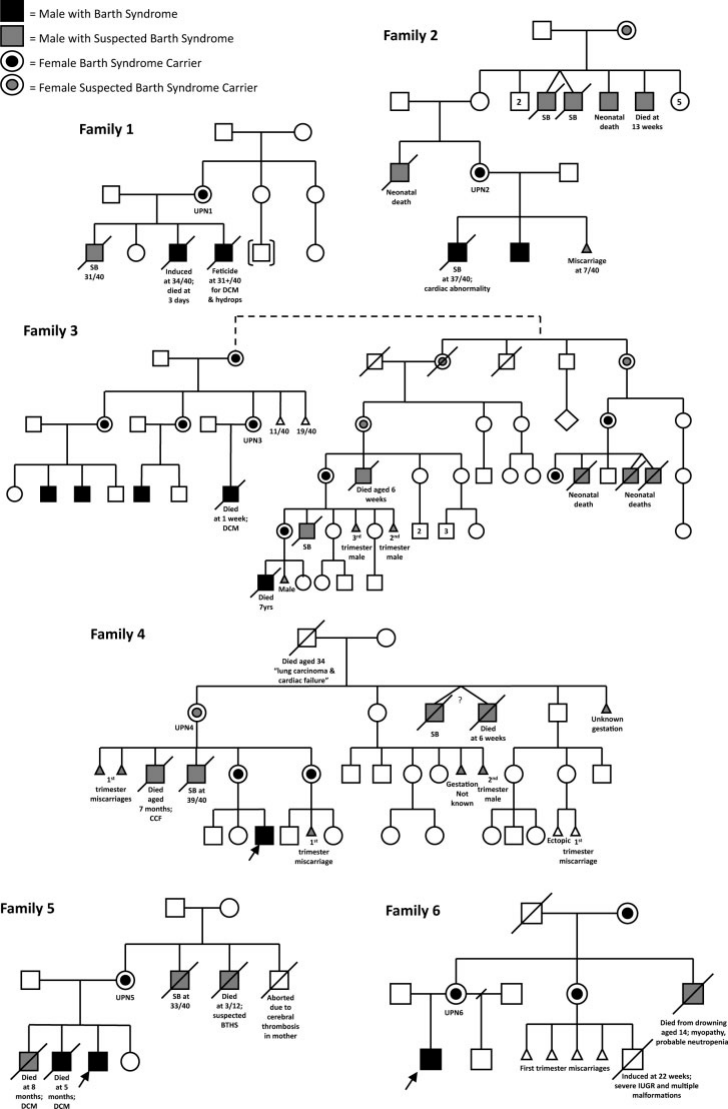
Pedigrees of families highlighting the high rate of late miscarriage and stillbirth

Six families taken from 19 unrelated kindreds with definitively proven BTHS had histories which included serious fetal or perinatal problems. Their family trees are shown in Figure 1. For ease of identification, each family is described with reference to one mother who is given a unique patient number (UPN). References to previous families found with the same mutations as patients described here are drawn from the Barth Syndrome Foundation genetic database.

In families 1, 2 and 3, severe cardiomyopathy (DCM, fetal hydrops or EFE) occurred in male fetuses subsequently shown to have *TAZ* mutations. Families 4 and 5 have suspicious histories of male third trimester fetal loss and/or stillbirth in addition to living boys diagnosed with BTHS. A proven carrier female in family 6 has had five fetal losses at up to 22 weeks' gestation. In these families, a total of 9 males were stillborn and 14 died as neonates or infants but there were no spontaneous abortions, stillbirths or childhood deaths in females.

### Family 1

The index mother's (UPN1) first pregnancy resulted in spontaneous loss of a stillborn male fetus at 31 weeks' gestation (birth weight 1.57 kg, approximately 30 weeks by measurements). His heart (weight 10.5 g) and body were considered to be anatomically normal, although extensive maceration and autolysis prevented determination of the cause of fetal demise. The second pregnancy resulted in delivery of a healthy normal female at term. Ultrasound examination at 31 weeks during a third pregnancy revealed a hydropic male with poor cardiac contractility. Delivery was induced at 34 weeks' gestation because of deteriorating fetal condition (birth weight 2.9 kg). He required intubation at birth and inotropic support for poor left ventricular (LV) function and dilatation, followed by drainage of pleural effusions and ascites. Following progressive deterioration, care was withdrawn on day 3 of life. Postmortem showed biventricular dilatation (most marked on the left), EFE, mild secondary lung hypoplasia, renal tubular/cortical and pontosubicular neuronal necrosis. The thymus was atrophic (2.7 g, expected weight 8 g) with marked lymphocyte depletion on histology. Cardiac histology demonstrated no features to enable a specific diagnosis. During a fourth pregnancy, feticide was performed at 31 + weeks after an antenatal diagnosis of cardiomyopathy and hydrops. At postmortem, the fetus weighed 2.32 kg with linear measurements equivalent to 37 weeks' gestation. There was biventricular cardiac dilatation with mild diffuse EFE but no focal myocardial lesions and no vacuolation or other features suggestive of metabolic disease on histology. The thymus was atrophic (2.5 g, expected weight 7 g). Skin fibroblasts grown from the second male showed a highly aberrant MLCL/CL ratio, and mutation analysis of DNA from the second and third males revealed a missense mutation in the *TAZ* gene (c.280C > G, p.Arg94Gly). This mutation has been previously reported as causing BTHS, affects a highly conserved residue, and is identified occurring *de novo* in the mother, providing good evidence for causation.

### Family 2

The index mother's (UPN2) first pregnancy resulted in an emergency lower segment caesarian section (LSCS) at term and delivery of a male neonate who required intubation at delivery. DCM was diagnosed, with an LV fractional shortening of 10%. Initial neutrophil count, white cell enzyme assays, serum lactate, amino acid/organic acid/oligosaccharide analysis were all normal, although neutropenia developed later. He required a long period of ventilation and aggressive inotropic support. Echocardiogram at 3 months suggested LVNC.

In her second pregnancy, cardiac ultrasound was normal at 22 weeks' gestation but a male fetus was stillborn at emergency caesarian section at 37 weeks following an abnormal cardiotocograph. Birth weight was 2.95 kg and crown-heel length 47 cm. Postmortem suggested that death had probably occurred several days prior to delivery. There were no dysmorphic features although the ears were large. The heart showed LV myocardial thickening (8 mm maximum thickness) and LV dilatation but normal right ventricular dimensions. Histology demonstrated vacuolated myocytes and biventricular EFE. T-associated areas of the spleen were reduced and the cortical thickness of the thymus markedly reduced with appreciable lymphocyte depletion. A mitochondrial cytopathy was suspected but further studies were prevented by autolysis of the cardiac tissue.

The cause of DCM in the firstborn was eventually shown to be BTHS by demonstration of a highly aberrant MLCL/CL ratio and demonstration of a c.583 + 5G > A mutation in IVS7 of the *TAZ* gene, a mutation predicted to lead to aberrant RNA splicing. The same mutation was subsequently confirmed in tissue from the stillborn fetus and UPN2 was confirmed as a carrier of the BTHS mutation. She subsequently had a further miscarriage at 7 weeks' gestation. The wider family history was also suspicious: UPN2's mother had a full-term male who died at 2 h of age and was said to have had a problem with his heart valves. Her grandmother had 12 babies from 11 pregnancies: six healthy girls, two healthy boys, twin males who were stillborn, a boy who died soon after birth and a further boy who died after 13 weeks in hospital.

### Family 3

This family has a very extensive history of BTHS due to a mutation in the *TAZ* gene (exon 8, c.589G > A, p.Gly197Arg; a mutation ‘hotspot’ which has been identified in several families, is evolutionarily highly conserved, and segregates with the condition in this family). UPN3 (a known carrier) gave birth to a male who developed DCM at 22 weeks' gestation and who was delivered by urgent LCSC at 32 weeks due to fetal distress. Although initially well controlled on inotropes, he died at 1 week due to cardiac decompensation triggered by ventricular arrhythmias. Disease expression in this family has been very variable. A cousin with BTHS was well until falling behind in achievement of motor milestones from 1 year. He developed feeding problems and lethargy at 2.5 years, was diagnosed with DCM at 3.5 years and required cardiac transplantation several months later. Another cousin was only diagnosed with BTHS after the disease had been identified in his brother (who developed DCM at 3 months of age, without accompanying neutropenia); when diagnosed at 3.5 years this boy's only sign was proximal myopathy. Another distant relative (previously reported in Ronghe *et al.*, 2001) died of post-transplant lymphoproliferative disease 7 years after the cardiac transplantation for DCM which presented at 6 months. His grandmother (a proven carrier of the familial *TAZ* mutation) had six pregnancies, which resulted in live birth of three normal females in addition to three male fetal deaths, one each in the second and third trimesters and one stillborn at term. Postmortems were not performed on these fetuses. A distant male relative (whose sister and mother are proven carriers of the G197R *TAZ* mutation) died of DCM with EFE at 4 months. This child's male cousin (whose mother is a proven carrier) died of heart disease at 6 months. Another male cousin was stillborn at term. There were two further male deaths at 4 months with cyanosis in the previous generation of this family.

### Family 4

The index mother, UPN4, had a brother who died at 6 weeks of age. He was one of male twins, the other being stillborn. UPN4 went on to have six pregnancies, including two first trimester miscarriages. Her first pregnancy produced a male child who died due to congestive cardiac failure with EFE at 7 months of age. Her third pregnancy resulted in a male stillbirth at 39 weeks' gestation (although no fetal movements were felt from 33 weeks). No autopsy details are available. Two subsequent pregnancies produced two normal females: the second son of one of these daughters developed DCM at 8 months: mutation analysis of DNA from this child revealed a missense mutation in the *TAZ* gene (exon 8, c.626T > A, p.Ile209Asn). This mutation has been previously reported to cause BTHS and is a highly conserved residue. This was confirmed in his mother and the other daughter of UPN4. The latter has a healthy son and daughter but is known to have had a first trimester miscarriage (cause unknown).

### Family 5

The firstborn male to mother UPN5 had intrauterine growth restriction (IUGR) and was born by spontaneous vaginal delivery (SVD) at 38 weeks' gestation weighing 2.4 kg. He failed to thrive and then died due to DCM at 7.5 months. Her second born male had IUGR and was born weighing 2.27 kg at 38 weeks' gestation. DCM was present from birth, presenting as feeding problems and failure to thrive, and led to his death at 5.5 months. The third pregnancy resulted in a male who was born at 32.5 weeks' gestation weighing 2.38 kg by induced delivery due to fetal distress. Heart failure was not present at birth but he began to sweat excessively during feeds in the early months of life, showed delayed motor development and hypotonia, and then developed DCM at 5 months. Cardiac transplantation was required at 1.5 years (previously reported in Mangat *et al.*, 2007). Muscle biopsy performed during investigation of his heart failure showed lipid storage myopathy, reduced cytochrome oxidase activity and reduced respiratory chain activity of complexes I, III and IV. The explanted heart also showed reduced activity of complexes I and IV. His motor and speech development were subsequently delayed and he developed significant signs of myopathy (e.g. unable to do up buttons at 5 years, positive Gower's sign at 10 years). He subsequently suffered many infections but is now, at 17 years, well controlled on anti-rejection drugs, prophylactic antibiotics and granulocyte colony-stimulating factor to alleviate neutropenia.

BTHS was subsequently proven by identification of a missense mutation in *TAZ* (exon 2, c.207C > G, p.His69Gln), which has been previously reported as causing BTHS and is a highly conserved residue, and by confirmation of an abnormal MLCL/CL ratio. UPN5 is a proven carrier of this mutation. Her mother lost two males: the first was stillborn at 33 weeks and the second died at 3 months of suspected fulminant viremia, having been listless and with feeding difficulties for some time prior to the acute episode. A third male fetus was aborted after the lady developed a cerebral thrombosis.

### Family 6

The second male born to UPN6 by full-term normal delivery weighing 3.2 kg developed grunting respiration, acidosis and hypoglycemia (2.2 mmol/L) at 3 days of age, leading to a diagnosis of DCM. He was found to be neutropenic with 3-MGC aciduria and a markedly deranged MLCL/CL ratio. Mutation analysis revealed a frameshift mutation in *TAZ* (exon 11; c.837_838delTC, p.Gln280GlyfsX30), which is predicted to be pathogenic, and was confirmed in his mother but not in his healthy brother. The brother of UPN6 had failure to thrive, proximal myopathy, motor delay (walked at 2.5–3 years), recurrent gingivitis and was reported to have had an ‘abnormal white blood count’ (no details available). He drowned at 14 years of age; cardiac appearance was reported as normal at postmortem. This child's maternal aunt, who is a proven mutation heterozygote, has had five fetal losses, four early in pregnancy and one at 22 weeks with multiple malformations.

## DISCUSSION

Cardonick *et al.* (1997) were the first authors to conclusively demonstrate fetal onset of cardiomyopathy in BTHS. They reported a mother with a history of DCM in three brothers. Her first child developed asymmetric IUGR, oligohydramnios and LV dysfunction by 33 weeks' gestation, having had a normal echocardiogram and growth parameters at 21 weeks *in utero*. Ventricular dysfunction was still present at birth after induction at 35 weeks and a diagnosis of BTHS was confirmed by the finding of the 3-MGC aciduria and neutropenia.

Subsequently, Brady *et al.* (2006) reported an Iranian first cousin couple with an extensive family history of male infant/toddler death whose first male child died at 10 months from a cardiomyopathy. This was believed to be secondary to a fatty acid oxidation or mitochondrial oxidative phosphorylation disorder (Brady *et al.*, 2006). In a subsequent pregnancy, ascites and cardiomegaly were detected in a male fetus at 32 weeks' gestation. That baby was delivered at 33 weeks by LSCS with growth parameters on the 25 to 50th centiles. Cardiac ejection fraction was 16%, but there was no excess organic aciduria and a normal white blood count. After death, at 12 days, autopsy revealed EFE accompanied by vacuolization of the subendocardial myocytes and enlarged mitochondria with disorganized cristae on electron microscopy of the myocardium. BTHS was confirmed by demonstration of a *TAZ* mutation.

In the current report, we demonstrate that the fetal cardiomyopathy associated with proven BTHS may result in fetal demise or early neonatal death. Two out of three male pregnancies to UPN1 developed hydrops by 31 weeks and a third was stillborn at the same point in gestation. This mother's first male fetus was too macerated to allow reliable autopsy conclusions. Her second was delivered early—at 34 weeks—because of hydrops secondary to cardiomyopathy, but this child still succumbed on day 3 of life despite aggressive management. The third male pregnancy was terminated at 31 weeks after the fetus became hydropic; DCM and diffuse EFE were demonstrated at autopsy. Mutations were subsequently confirmed in her second and third male fetuses and UPN1 herself was confirmed as a carrier of a *TAZ* mutation. The first male fetus born to UPN2 is thought to have died *in utero* at 36 to 37 weeks secondary to DCM with EFE. BTHS was subsequently proven in her next male pregnancy and UPN2 is a proven carrier. The male fetus born to UPN3 developed DCM at 22 weeks and required urgent delivery at 32 weeks but died due to cardiac decompensation at 1 week.

The male fetal losses and neonatal deaths in families 4, 5 and 6 were not accompanied by postmortem examination. However, there is no history of female miscarriage, stillbirth or neonatal death in these families. As a *TAZ* mutation has been confirmed in each of these families, we suggest that these deaths are highly likely to have been the result of BTHS.

It is not clear to what extent (if at all) BTHS has contributed to the five miscarriages in UPN6, a known carrier. However, Brady *et al.* (2006) showed that cardiomegaly, EFE and subendocardial vacuolization of the myocytes can be present as early as 18 weeks *in utero* in a fetus subsequently proven to have BTHS (who was aborted electively at that gestation). One of the fetuses of the sister of UPN6 was spontaneously aborted at 22 weeks' gestation with multiple malformations. This may have resulted from an alternative condition, e.g. chromosome abnormality. However, facial dysmorphism is recognized in BTHS (Hastings *et al.*, 2009) and we cannot exclude more significant congenital anomalies as a further manifestation of the disorder.

While cardiac failure *per se* has undoubtedly contributed to the deaths reported in this and other publications, it is impossible to rule out the possibility that ventricular arrhythmia or mitochondrial dysfunction were significant contributory factors. For example, Yen *et al.* (2008) observed acute metabolic decompensation in a 13-day-old child who developed respiratory failure within 8 h of presenting with lactic acidosis, hyperammonemia, hypoglycemia and coagulopathy. Donati *et al.* (2006) noted similar metabolic changes in 1- and 3-day-old babies. We have also seen a child from another family with proven BTHS who presented with severe lactic acidosis and hypoglycemia on the first day of life despite absence of cardiac problems (although DCM did develop by 19 months).

There was no evidence of fetal loss in 13 out of 19 unique families diagnosed in the UK to date. The mutations in all 19 families were evaluated for evidence of possible genotype/phenotype correlation. The six families described in this report are unrelated, and have unique individual mutations. Four produce amino acid substitutions within conserved motifs (families UPN1, 3, 4 and 5). One family has a splice site mutation that would be predicted to result in a truncated protein (UPN2). One family has a frameshift mutation in the last exon that results in an extension of the protein (UPN6). BTHS is known for its phenotypic variability even within sibships, as highlighted by the cousins described in Family 3.

It should also be noted that no female carrier of BTHS has ever been shown to have symptomatology related to their carrier state. Only 12% of mothers tested do not carry their son's mutation; moreover, a limited study of pedigrees of carrier mothers showed that 8 out of 11 had inherited a *de novo* mutation from a parent or grandparent (Kirwin *et al.*, 2007). This means that fetuses may be lost to such mothers and BTHS would not be suspected because of the absence of a family history.

## CONCLUSION

We suggest that BTHS is an underrecognized cause of male fetal demise that may present as a number of common obstetric scenarios: unexplained pregnancy loss, hydrops or DCM + /− EFE. MLCL/CL testing provides a sensitive diagnostic test in fresh or stored fetal material, or neonatal blood spots, of boys who have died from problems suggestive of BTHS and for families where suspicious multiple male fetal/neonatal deaths have occurred. Mutation testing of the *TAZ* gene in families identified through MLCL/CL testing allows genetic prenatal diagnosis in subsequent pregnancies. Unfortunately, there is no abnormality of the MLCL/CL ratio in heterozygous females so that mothers are not open to simple screening where fetuses have been lost previously (Kulik *et al.*, 2008).
